# The Effect of Obesity on Response to Neoadjuvant Therapy in Locally Advanced Gastric Cancer

**DOI:** 10.31557/APJCP.2020.21.9.2723

**Published:** 2020-09

**Authors:** Aysegul Sakin, Suleyman Sahin, Abdullah Sakin, Mehmet Naci Aldemir, Irfan Bayram, Cetin Kotan

**Affiliations:** 1 *Department of Internal medicine, University of Health Sciences, Van Research and Training Hospital, Van, Turkey. *; 2 *Department of Medical Oncology, University of Health Sciences, Van Research and Training Hospital, Van, Turkey.*; 3 *Department of Medical Oncology, Yuzuncu Yil University Medical School, 65030, Van, Turkey. *; 4 *Department of Pathology, Yuzuncu Yil University Medical School, 65030, Van, Turkey. *; 5 *Department of General surgery, Yuzuncu Yil University Medical School, 65030, Van, Turkey. *

**Keywords:** Obesity, gastric cancer, tumor regression grade, body mass index

## Abstract

**Introduction:**

The effect of obesity on response to neoadjuvant chemotherapy (NACT) remains unknown. We aimed to investigate the effect of obesity on response to NACT and survival in locally-advanced gastric cancer (GC).

**Methods:**

From 2010 to 2019, 142 GC patients with clinical stage III disease who underwent curative surgery after NACT were enrolled. Patients were divided into 3 groups according to body mass index (BMI) as follows; BMI < 25 kg/m^2^, BMI = 25-30 kg/m^2^, and BMI > 30 kg/m^2^. The Mandard tumor regression grading system was used for tumor regression grade (TRG).

**Results:**

Of the 142 GC patients, 45(31.7%) were female. The median age was 58 years. BMI was < 25 kg/m^2^ in 60 (42.3%) patients, 25-30 kg/m2 in 44 (31%) patients, and > 30kg/m^2^ in 38 (26.8%) patients. The numbers of patients with TRGI-II, TRGIII, and TRGIV-V were 35 (24.6%), 44 (31%), and 63 (44.4%), respectively. There was no statistically significant difference among BMI groups in terms of disease-free survival (DFS) and overall survival (OS) (p = 0.919 and p = 0.398, respectively). According to TRG groups; mDFS was 46 months in TRG I-II, 28 months in TRG III, and 18 months in TRG IV-V (p<0.001). In multivariate analysis, presence of perineural invasion and lymphovascular invasion were the factors affecting TRG.

**Conclusion:**

In our study, we found that pre-treatment obesity did not affect the TRG in clinical stage III GC patients. However, a better TRG status was associated with improved survival.

## Introduction

Gastric cancer (GC) remains a major cause of cancer-related deaths globally, with high mortality rates even in the early stages. In population-based series, the 5-year survival rate for completely-resected stage I-II GC patients is approximately 35-75% (Ferlay et al., 2015; Siegel et al., 2019). Multidisciplinary team approach is the standard of care in the treatment of GC. Randomized trials and meta-analyses have indicated a significant survival benefit with adjuvant chemoradiotherapy (CRT), Neoadjuvant chemotherapy (NACT), and adjuvant chemotherapy (ACT) for locally-advanced GC patients, as compared with surgery alone (Cunningham et al., 2006; Xiong et al., 2014; Al-Batran et al., 2019).

NACT prior to surgery can prompt tumor shrinkage, decrease intraoperative spread, and increase the rate of R0 resection during surgery. Neoadjuvant treatment evaluates the effect of NACT regimen as well as guiding the postoperative treatment approach. Moreover, if metastatic spread occurs during or after NACT, particularly in patients who have a greater risk of developing distant metastases, an unnecessary surgery will be prevented .The responses to frequently-used chemotherapy (CT) regimens range from 49% to 69.7% (Kobayashi and Kimura, 2000; Cunningham et al., 2006).

The rate of (pathological complete response) pCR in GC after NACT is relatively low. Previous studies and meta-analysis have reported that pCR increases survival (Lorenzen et al., 2013; Li et al., 2018). However, it is difficult to define which patient effectively responds to NACT. The ability to predict the pathological tumor response before treatment can provide a significant clinical advantage, provide additional information to allow tailored ACT options, and help evaluate the individual prognosis (Melcher et al., 1996; Li et al., 2012; Al-Batran et al., 2016).

Obesity is an increasing global health problem. Body mass index (BMI), calculated by the patient’s weight and height, is a good way to measure obesity. Moreover, BMI is an effective method in evaluating the nutritional status of cancer patients (Liedman et al., 1996; Mokdad et al., 2003). Many studies have shown that obesity is associated with poor surgical outcomes in cancer patients, including GC (Bege et al., 2009; Benns et al., 2009; Kunisaki et al., 2009).

In previous studies, the relation of obesity with postoperative complications and survival was examined (Dhar et al., 2000; Tsujinaka et al., 2007; Kunisaki et al., 2009; Kunisaki, 2010). However, the effect of obesity on response to NACT remains unknown. In the present study, we aim to investigate the effect of obesity on response to treatment and long-term survival in clinical stage III GC patients treated with NACT.

## Materials and Methods

*Study population*

From 2010 through 2019, all patients with locally-advanced GC, who underwent NACT followed by gastrectomy in Van Yüzüncü yıl University Hospital, were analyzed retrospectively. The inclusion criteria were defined as follows; age ≥ 18 years and having received NACT for locally-advanced (clinical stage III) GC. Patients with any of the following criteria were excluded from the study; age <18 years, those not undergoing surgery, metastatic disease, history of a second primary cancer, histologic subtypes other than adenocarcinoma (AC), [e.g., Signet ring cell carcinoma (SRCC), and Mucinous adenocarcinoma (MAC)], patients who died due to surgical complications, and those with missing data. The clinical stage of the patients was determined by computed tomography taken before treatment. Patients were restaged according to the AJCC (American Joint Committee on Cancer) Cancer Staging Manual, 8th edition.

*Data collection*

Demographic data of the patients including gender, age, Eastern Cooperative Oncology Group performance scale (ECOG PS), height, weight, presence of hypertension (HT) or diabetes mellitus (DM), smoking status, clinical stage, Lauren classification, primary tumor localization, histology (AC, MAC, and SRCC), tumor grade, neoadjuvant regimen, type of surgery (subtotal, total gastrectomy, D1, or D2 dissection), ypTNM stage, pathological tumor stage (ypT), pathological lymph node stage (ypN), presence of lymphovascular (LVI) and perineural invasion (PNI), tumor regression grade(TRG), human epidermal growth factor receptor 2 (HER-2) status, adjuvant regimen, recurrence status, site of recurrence, and final status were obtained from the written archive files. Patients were divided into 3 groups according to BMI as follows; BMI < 25 kg/m^2^, BMI = 25-30 kg/m^2^, and BMI > 30 kg/m^2^. In this study, no classification such as BMI <18.5 kg/m^2 ^as a separate group could be made since there were only 3 patients with BMI < 18.5 kg/m^2^.

*Response to treatment*

The Mandard tumor regression grading system was used for TRG, which was defined as follows; TRG I= Complete regression, fibrosis with no evidence of tumor cells in the specimen, TRG II= Fibrosis and rare residual tumor cells in the specimen, TRG II= Fibrosis outgrowing residual tumor in the specimen, TRG IV= Rare fibrosis and residual tumor outgrowing fibrosis, and TRG V= Tumor without evidence of regressive changes. The patients were divided into 2 groups according to TRG status; Group 1= TRG I-II and group 2= TRG III-IV-V. 

*Followup*

Disease-free survival (DFS) was calculated from the date of diagnosis to the date of progression or last follow-up. Overall survival (OS) was calculated from the date of diagnosis to the date of death or last follow-up.

*Ethics committee approval*

This study was conducted in accordance with the Declaration of Helsinki and it was reviewed and approved by the Ethics Committee of the Van Yüzüncü Yıl University Faculty of Medicine (2020/03-52).

*Statistical analysis*

Statistical Package for Social Sciences 22.0 for Windows software (Armonk NY, IBM Corp. 2013) was used for all statistical analysis. Student’s t test was used when the numerical variable provided the normal distribution condition in two independent groups, whereas Mann Whitney U test was used when the normal distribution condition was not provided. Chi-square analysis was used to compare the ratios in the groups. Survival analyzes were performed by Kaplan Meier Analysis. For the determinant factors, logistic regression analysis was used. Statistical significance level was accepted as p <0.05.

## Results

*Clinicopathological characteristics*

Of the 142 GC patients, 45 (31.7%) were female and 97 (68.3%) were male. The median age was 58 years (range, 31-79). BMI was < 25kg/m^2^ in 60 (42.3%) patients, 25-30 kg/m^2^ in 44 (31%) patients, and >30 kg/m^2^ in 38 (26.8%) patients. Twelve (8.5%) patients had DM and 26 (18.3%) patients had HT. Sixty-nine (48.6%) patients were smokers. According to the Lauren classification, tumor was intestinal type in 120 (84.5%) patients. In 101 (71.1%) patients, histological subtype was AC. The tumor grade in 41 (29.3%) patients was 3. As a surgical procedure, 119 (83.8%) patients underwent total gastrectomy and 94 (66.2%) patients had D1 dissection. PNI was present in 77 (54.2%) patients and LVI was positive in 66 (46.5%) patients. During the median follow-up time of 15 months, 52 (36.6%) patients developed recurrence and 23 (16.2%) patients died (Table 1).

*Treatment regimens*

Considering the NACT regimens, 52 (36.6%) patients received either epirubicin + oxaliplatine + capecitabine (EOX), epirubicin + oxaliplatine + fluorouracil (EOF), epirubicin + cisplatin + capecitabine (ECX), or epirubicin + cisplatin + fluorouracil (ECF), 27 (19%) patients received either docetaxel + cisplatin + fluorouracil (DCF) or docetaxel + cisplatin + capecitabine (DCX), and 63 (44.4%) patients received fluorouracil + folinic acid + oxaliplatine + docetaxel (FLOT). Patients received an average of 3.5 ±1.4 CT cycles. All patients were able to receive ACT. As adjuvant regimens; 35 (24.6%) patients were given either capecitabine + oxaliplatine (XELOX), fluorouracil + folinic acid + oxaliplatine (FOLFOX), or fluorouracil + cisplatin (CF), 43 (30.3%) patients were given either EOX, EOF, ECX, or ECF, 21 (14.8%) patients were given either DCF or DCX, and 43 patients were given FLOT (Table 1).

*Clinicopathological features in TRG groups*

Thirty-five (24.6%) patients were TRG I-II, 44 (31%) patients were TRG III, and 63 (44.4%) patients were TRG IV-V. There was no significant difference between the TRG groups in terms of age, gender, comorbidity, Lauren classification, tumor localization, tumor grade, NACT regimen, the numbers of NACT cycles, surgical margin, the numbers of lymph nodes removed, HER-2 status, and ACT regimen. There was a statistically significant difference among the groups in terms of ypTNM, ypT, ypN, the number of lymph nodes removed, LVI, PNI, development of recurrence, and exitus rates (Table 2).

*Survival analysis*

**Figure 1.**
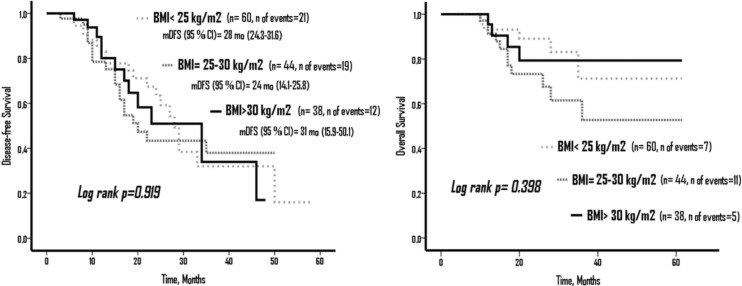
Disease-Free Survival (DFS) and Overall Survival (OS) According to Body Mass Index (BMI) Groups

According to BMI groups, there was no statistically significant difference in terms of DFS and OS (p=0.919 and p=0.398, respectively). mDFS durations in BMI < 25 mg/kg^2^, BMI = 25-30 mg/kg^2^, and BMI > 30 mg/kg^2^ were 28 months (95% confident interval [CI], 24.3-31.6), 24 months (95%CI, 14.1-25.8), and 31 months (95% CI, 15.9-50.1), respectively. However, mOS could not be reached (Figure 1)

**Figure 2:**
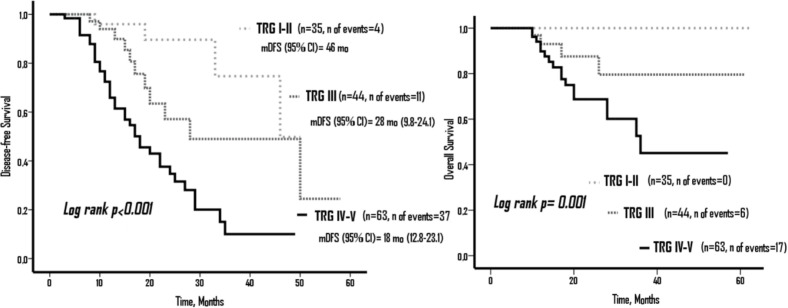
Disease-Free Survival (DFS) and Overall Survival (OS) According to Tumor Regression Grade (TRG) Groups

Based on the TRG groups, there was a statistically significant difference in terms of DFS and OS (p<0.001 and p=0.001, respectively). According to TRG groups; mDFS was 46 months in TRG I-II, 28 months (95% CI, 9.8-24.1) in TRG III, and 18 months (95% CI, 12.8-23.1) in TRGIV-V. However, mOS could not be reached (Figure 2).

*Factors affecting TRG*

In multivariate analysis with enter model; height, weight, BMI, Lauren classification, histology, and the number of NACT cycles did not affect TRG. However, presence of PNI (Odds ratio[OR],5.3, 95 % CI, 1.1-23.6) and LVI (OR, 25.0, 95 % CI, 4.3-144.4) affected TRG (p=0.028 and p<0.001, respectively) (Table 3). 

**Table 1:** Patient Characteristics and Clinicopathological Features in BMI Groups

Variable		Total Patients (n=142)		BMI<25 kg/m^2 ^(n=60)		BMI=25-30 kg/m^2^(n=42)		BMI>30 kg/m^2 ^(n=38)		p
		n	%	n	%	n	%	n	%	
Gender	Female	45	31.7	13	21.7	12	27.3	20	52.6	0.004
	Male	97	68.3	47	78.3	32	72.7	18	47.4	
Age (year)	Median	58		60		57		59		0.192
	(min-max)	(31-79)		(21-79)		(44-77)		(42-71)		
ECOG PS	0	125	88	51	85	39	88.6	35	92.1	0.566
	1	17	12	9	15	5	11.4	3	7.9	
Height	cm	165.2±9.1		167.5±8.2		166.0±9.0		159.6±8.6		0.001
Weight	kg	71.5±12.5		61.1±9.1		73.7±7.9		82.5±9.8		<0.001
BMI	Kg/m^2^	26.29±4.71		21.6±2.3		26.9±1.0		32.2±2.1		<0.001
Hypertension	Yes	26	18.3	14	23.3	7	15.9	5	13.2	0.395
Diabetes mellitus	Yes	12	8.5	3	5	4	9.1	5	13.2	0.381
Smoking status	Yes	69	48.6	34	56.7	21	47.7	14	36.8	0.159
Lauren classification	Intestinal	120	84.5	46	76.7	38	86.4	36	94.7	0.051
	Diffuse	22	15.5	14	23.3	6	13.6	2	5.3	
Location	GEJ	8	5.6	6	10	0	0	2	5.3	0.101
	Cardia	61	43.0	27	45	17	38.6	17	44.7	
	Body	32	22.5	15	25	11	25	6	15.8	
	Antrum	37	26.1	9	15	16	36.4	12	31.6	
	Linitis plastica	4	2.8	3	5	0	0	1	2.6	
Histology	SRCC	24	16.9	11	18.3	9	20.5	4	10.5	0.738
	AC	101	71.1	42	70	29	65.9	30	78.9	
	MAC	17	12.0	7	11.7	6	13.6	4	10.5	
Grade	I	12	8.6	3	5.2	4	9.1	5	13.2	0.68
	II	87	62.1	39	67.2	26	59.1	22	57.9	
	III	41	29.3	16	27.6	14	31.8	11	28.9	
NACT regimen	ECF-ECX- EOF-EOX	52	36.6	14	23.3	15	34.1	23	60.5	0.01
	DCF-DCX	27	19.0	10	16.7	13	29.5	4	10.5	
	FLOT	63	44.4	36	60	16	36.4	11	28.9	
NACT cycle no.		3.8±1.4		3.7±1.1		3.9±1.1		3.7±2.3		0.082
Gastrectomy	Subtotal	23	16.2	7	11.7	9	20.5	7	18.4	0.422
	Total	119	83.8	53	88.3	35	79.5	31	81.6	
Lymphadenectomy	D1	94	66.2	40	66.7	32	72.7	22	57.9	0.365
	D2	48	33.8	20	33.3	12	27.3	16	42.1	
Surgical margin	Positive	132	93.0	53	88.3	42	95.5	37	97.4	0.218
	Positive	10	7.0	7	11.7	2	4.5	1	2.6	
ypTNM	0	12	8.5	4	6.7	5	11.4	3	7.9	0.776
	1	18	12.7	7	11.7	6	13.6	5	13.2	
	2	36	25.4	14	23.3	9	20.5	13	34.2	
	3	76	53.5	35	58.3	24	54.5	17	44.7	
ypT	0	12	8.5	4	6.7	5	11.4	3	7.9	0.722
	1	17	12.0	5	8.3	5	11.4	7	18.4	
	2	11	7.7	5	8.3	3	6.8	3	7.9	
	3	70	49.3	31	51.7	19	43.2	20	52.6	
	4	32	22.5	15	25	12	27.3	5	13.2	
ypN	0	45	31.7	19	31.7	15	34.1	11	28.9	0.423
	1	30	21.1	10	16.7	10	22.7	10	26.3	
	2	22	15.5	9	15	4	9.1	9	23.7	
	3	45	31.7	22	36.7	15	34.1	8	21.1	
No. of nodes removed		27.3±13.1 (9-78)		24.6±11.4		26.6±11.2		31.4±15.0		0.13
No. of nodes positive		5.05±6.90 (0-35)		5.1±6.1		5.2±7.9		4.1±5.4		0.703
PNI	Presence	77	54.2	31	51.7	25	56.8	21	55.3	0.869
Variable		Total Patients (n=142)		BMI<25 kg/m^2 ^(n=60)		BMI=25-30 kg/m^2^(n=42)		BMI>30 kg/m^2 ^(n=38)		p
		n	%	n	%	n	%	n	%	
LVI	Presence	66	46.5	29	48.3	20	45.5	17	44.7	0.929
HER-2 status	0	108	76.1	43	71.7	36	81.8	29	76.3	0.752
	1	12	8.5	6	10	3	6.8	3	7.9	
	2	12	8.5	6	10	4	9.1	2	5.3	
	3	10	7	5	8.3	1	2.3	4	10.5	
TRG	I	12	8.5	4	6.7	5	11.4	3	7.9	0.962
	II	23	16.2	10	16.7	6	13.6	7	18.4	
	III	44	31	17	28.3	13	29.5	14	36.8	
	IV	58	40.8	27	45	18	40.9	13	34.2	
	V	5	3.5	2	3.3	2	4.5	1	2.6	
TRG groups	Group 1	35	26.3	14	23.3	11	25	10	26.3	0.944
	Group 2	107	73.7	46	76.7	33	75	28	73.6	
ACT regimen	XELOX-FOLFOX-CF	35	24.6	19	31.7	10	22.7	6	15.8	0.045
	EOX-EOF-ECX-ECF	43	30.3	12	20	12	27.3	19	50	
	DCF-DCX	21	14.8	7	11.7	9	20.5	5	13.2	
	FLOT	43	30.3	22	36.7	13	29.5	8	21.1	
Recurrence and localization	Yes	52	36.6	21	35	19	43.2	12	31.6	0.552
	Locoregional	2	3.8	0	0	1	5.3	1	8.3	0.281
	Liver	16	30.8	5	23.8	7	36.8	4	33.3	
	Peritoneum	20	38.5	7	33.3	8	42.1	5	41.7	
	Distant Ln	6	11.5	2	9.5	2	10.5	2	16.7	
	Lung	5	9.6	5	23.8	0	0	0	0	
	Brain	1	1.9	0	0	1	5.3	0	0	
	Bone	2	3.8	2	9.5	0	0	0	0	
Last Status	Exitus	23	16.2	7	11.7	11	25	5	13.2	0.159
										

Abbreviations: AC, Adenocarcinoma; ACT, Adjuvant chemotherapy; BMI, Body mass index; CF, Fluorouracil + cisplatin; ECOG PS, Eastern cooperative oncology group performance scale; DCF, Docetaxel + cisplatin + fluorouracil; DCX, Docetaxel + cisplatin + capecitabine; ECF, Epirubicin + cisplatin + fluorouracil; ECX, Epirubicin + cisplatin + capecitabine; EOF, Epirubicin + oxaliplatin + fluorouracil; EOX, Epirubicin + oxaliplatin + capecitabine; FLOT, Fluorouracil + folinic acid + oxaliplatin + docetaxel; FOLFOX, Fluorouracil + folinic acid + oxaliplatin; GEJ, Gastroesophageal junction; HER-2, Human epidermal growth factor receptor 2; PNI, Perineural invasion; SRCC, Signet ring cell carcinoma; LN, Lymph node; LVI, Lymphovascular invasion; MAC, Mucinous adenocarcinoma; NACT, Neoadjuvant chemotherapy; TRG, Tumor regression grade; XELOX, Capecitabine + oxaliplatin; ypT, Pathological tumor stage; ypN, Pathological lymph node stage.

**Table 2:** Univariate Analysis for TRG Groups (TRG I-II vs. III-IV-V).

Variable			Group 1 (n=35)				Group 2 (n=107)					p	
			n		%		n		%				
Gender	Female		7		20		38		35.5			0.87	
	Male		28		80		69		64.5				
Age (year)	Median		61				58					0.192	
	(min-max)		(35-79)				(31-75)						
ECOG PS	0		33		94.3		92		86			0.242	
	1		2		5.7		15		14				
Height	cm		168.09±9.42				164.21±8.89					0.033	
Weight	kg		73.74±13.90				70.77±12.08					0.24	
BMI	Kg/m^2^		26.22±5.42				26.32±4.46					0.916	
Hypertension	Yes		7		20		19		17.8			0.766	
Diabetes mellitus	Yes		4		11.4		8		7.5			0.49	
Smoking status	Yes		19		54.3		50		46.7			0.437	
Lauren classification	Intestinal		32		91.4		88		82.2			0.192	
	Diffuse		3		8.6		19		17.8				
Location	GEJ		4		11.4		4		3.7			0.511	
	Cardia		14		40		47		43.9				
	Body		8		22.9		24		22.4				
	Antrum		9		25.7		28		26.2				
	Linitis plastica		0		0		4		3.7				
Histology	SRCC		7		20		17		15.9			0.151	
	AC		27		77.1		74		69.2				
	MAC		1		2.9		16		15				
Grade	I		4		11.4		8		7.6			0.336	
	II		24		68.6		63		60				
	III		7		20		34		32.4				
NACT Regimen	EOF-EOX-ECF-ECX		12		34.3		40		37.4			0.797	
	DCX-DCF		8		22.9		19		17.8				
	FLOT		15		42.9		48		44.9				
NACT cycle no.			3.8±2.2				3.4±1.12					0.17	
Gastrectomy	Subtotal		7		20		16		15			0.482	
	Total		28		80		91		85				
Lymphadenectomy	D1		26		74.3		68		63.6			0.244	
	D2		9		25.7		39		36.4				
Surgical margin	positive		0		0		10		9.3			0.061	
ypTNM	0		12		34.3		0		0			<0.001	
	1		16		45.7		2		1.9				
	2		6		17.1		30		28				
	3		1		2.9		75		70.1				
ypT	0		12		34.3		0		0			<0.001	
	1		11		31.4		6		5.6				
	2		6		17.1		5		4.7				
	3		6		17.1		64		59.8				
	4		0		0		32		29.9				
ypN	0		32		91.4		13		12.1			<0.001	
	1		1		2.9		29		27.1				
	2		2		5.7		20		18.7				
	3		0		0		45		42.1				
No. of nodes removed			23.6±10.2 (9-51)				28.6 ±13.8(10-78)					0.08	
Variable				Group 1 (n=35)				Group 2 (n=107)			p	
				n		%		n		%		
No. Of nodes positive				0.26±0.93 (0-5)				6.66±7.28 (0-35)			<0.001	
PNI		Presence		5		14.3		72		67.3	<0.001	
LVI		Presence		2		5.7		74		69.2	<0.001	
HER-2 status		0		25		71.4		83		77.6	0.468	
		1		3		8.6		9		8.4		
		2		5		14.3		7		6.5		
		3		2		5.7		8		7.5		
TRG		I		12		34.3		0		0	<0.001	
		II		23		65.7		0		0		
		III		0		0		44		41.1		
		IV		0		0		58		54.2		
		V		0		0		5		4.7		
ACT regimen		XELOX-FOLFOX-CF		5		14.3		30		28	0.401	
		EOX-EOF-ECX-ECF		11		31.4		32		29.9		
		DCF-DCX		6		17.1		15		14		
		FLOT		13		37.1		30		28		
Recurrence and localization		yes		4		11.4		48		44.9	<0.001	
		locoregional		0		0		2		4.2		
		liver		1		25		15		31.3		
		Peritoneum		0		0		20		41.7		
		Distant LN		2		50		4		8.3		
		Lung		1		25		4		8.3		
		Brain		0		0		1		2.1		
		bone		0		0		2		4.2		
Last Status		Exitus		0		0		23		21.5	0.003	

Abbreviations: See Table 1.

**Table 3:** Multivariate Analysis for TRG Groups (TRG I-II vs. III-IV-V)

Variable		OR	95% CI for OR	p
Age	year	0.975	0.916-1038	0.427
Height	cm	0.897	0.800-1004	0.058
Weight	kg	1.023	0.922-1.134	0.667
BMI	<25kg/m^2^ (ref.)			0.369
	25-30 kg/m^2^	0.61	0.085-4.362	0.623
	>25kg/m^2^	0.125	0.004-3.617	0.226
Lauren classification	Diffuse vs Intestinal	1.423	0.210-9.600	0.717
Histology	SRCC(Ref.)	1		0.314
	AC	3.734	0.653-21.332	0.138
	MAC	4.384	0.250-76.750	0.312
NACT cycle no.		0.856	0.605-1.210	0.379
PNI	positive vs Negative	5.318	1.194-23.681	0.028
LVI	positive vs Negative	25.098	4.361-144.416	<0.001

Abbreviations: See Table 1.

## Discussion

In this study, we investigated the effect of obesity on response to NACT and long-term survival in clinical stage III GC patients. In addition, we evaluated the prognostic effect of TRG in GC patients with real life data and found that BMI did not affect both TRG and long-term survival. However, we observed that survival was significantly better in those with an increased response to NACT. In addition, the presence of PNI and LVI were determined as the predictive factors affecting TRG.

Previous studies have reported that the obesity affects the response to NACT in various solid tumors such as prostate cancer, rectal cancer, breast cancer, and pancreas cancer (Farr et al., 2017; Park et al., 2017; Duconseil et al., 2019; Sun et al., 2020). Park et al., (2017) reported that obesity reduced the complete response rate by 40% in their study with rectal cancer patients receiving neoadjuvant CRT. Likewise, another study of pancreatic cancer by Duconseil et al., (2019) has reported that obesity is determined as the factor affecting survival. Similarly, Karatas et al., (2017) has reported that obesity is an independent prognostic factor for pCR, with a poor survival in breast cancer patients who received NACT. 

Previous studies regarding GC patients have only investigated the effects of obesity on either post-surgical complications or mortality (Dhar et al., 2000; Kunisaki et al., 2009; Bickenbach et al., 2013; Wong et al., 2014; Palmela et al., 2017). Some of these studies also examined the relationship between obesity and long-term survival (Bickenbach et al., 2013; Wong et al., 2014). Wong et al. reported that obesity may cause technical difficulties to achieving R0 resection during gastric cancer surgery. However, an increased BMI did not affect DFS or OS (Wong et al., 2014). A study from Memorial Sloan-Kettering Cancer Center conducted by Bickenbach et al. evaluated the impact of obesity on survival in GC patients and reported fewer lymph node dissection rates and higher complication rates in patients with BMI > 25 kg/m^2^; however, survival was similar between the BMI groups (Bickenbach et al., 2013). In our study, patients who died due to surgical complications were not included. Furthermore, in our study, the ratio of number of positive lymph nodes to the total number of lymph nodes removed was similar between the BMI groups. In our study, BMI did not affect TRG. Similar to previous studies, we observed that BMI did not affect DFS and OS.

In the study with 264 GC patients by Xu et al., it was reported that TRG was correlated with both DFS and OS. Lauren classification and ypT were the independent factors for TRG (Xu et al., 2019). Zhu et al. reported that Mandard TRG system had correlation with survival in GC patients treated with NACT. In their study, TRG systems were significantly correlated with tumor grade, stage, LVI, PNI, and tumor size (Zhu et al., 2017). Smyth EC et al. conducted a randomized trial with GC patients and reported that TRG was in correlation with survival. In this study, mOS could not be reached in patients with TRG I-II, whereas mOS was 20 months in patients with TRG III-IV-V. Additionally, pathologic response to NACT was not correlated with any clinicopathological variable, including sex, age, tumor location, or histologic type (Smyth et al., 2016). Similar to these studies, in our study, TRG was correlated with survival. In our study, mDFS was 46 months in TRG I-II, while it was 18 months in TRG IV-V. Likewise, the TRG response was correlated with OS. Moreover, the presence of PNI and LVI were determined as the factors affecting TRG in our study.

In the literature, we could not find any study that examined the effect of BMI on TRG. Unlike the other studies, our study included a more heterogeneous patient group. We included only clinical stage III patients in our study. However, there were some limitations in our study. Our study was single-centered and had a retrospective nature. Since there were only 3 patients with BMI <18 in our study, they were not evaluated as a separate group. 

In conclusion, we found that pre-treatment obesity status did not affect the response to NACT or long-term survival in clinical stage III GC patients. However, presence of PNI and LVI were determined as the factors negatively affecting response to treatment. In our study, DFS and OS were significantly greater as the response to NACT increased.

*Main Points*

- The relation of obesity with postoperative complications and survival in solid cancers were examined in many studies

- The effect of obesity on response to neoadjuvant chemotherapy(NACT) in Gastric cancer(GC) remains unknown.

- In the study, we found that pre-treatment obesity status did not affect the response to NACT or long-term survival in clinical stage III GC patients.

- Presence of perineural invasion and lymphovascular invasion affected the response to NACT.

- Survival was significantly greater as the response to NACT increased
